# Examining Menthol Preference as a Correlate of Change in Cigarette Smoking Behavior over a One-Year Period

**DOI:** 10.3390/ijerph182010878

**Published:** 2021-10-16

**Authors:** Danielle R. Davis, Maria A. Parker, Cristine D. Delnevo, Andrea C. Villanti

**Affiliations:** 1 Vermont Center on Behavior & Health, Departments of Psychology & Psychiatry, University of Vermont, Burlington, VT 05405, USA; andrea.villanti@uvm.edu; 2 Department of Psychiatry, Yale School of Medicine, New Haven, CT 06511, USA; 3 Department of Epidemiology and Biostatistics, University of Indiana, Bloomington, IN 47405, USA; map2@iu.edu; 4 Center for Tobacco Studies, Rutgers Biomedical Health Sciences, New Brunswick, NJ 08854, USA; delnevo@cts.rutgers.edu; 5 Department of Health Behavior, Society & Policy, Rutgers School of Public Health, New Brunswick, NJ 08854, USA

**Keywords:** menthol, cigarette smoking, smoking changes, racial differences

## Abstract

Menthol cigarette use has been shown to be a contributing factor in the changes in smoking over time among youth. The current study aim was to use prospective survey data to identify if menthol cigarette use was associated with changes in smoking among adults. A representative cohort from the 2010 U.S. Tobacco Use Supplement to the Current Population Survey was interviewed at two time points one year apart. Respondents were past-30-day cigarette smokers at Wave 1 or Wave 2 categorized by menthol vs. non-menthol flavor preference (*n* = 3668). Trajectories were categorized as maintained, increased, or decreased smoking behavior between Waves. Multinomial logistic regressions examined if menthol cigarette use was associated with an increase/decrease in smoking behavior, adjusting for age, race/ethnicity, and sex. Menthol cigarette use was not associated with change over time in cigarette smoking in adult smokers. Age, race/ethnicity and sex were associated with changes in cigarette smoking. Young (vs. older) adults were more likely to increase smoking. Black and Hispanic smokers (vs. white smokers) were more likely to report any change in smoking. Males were less likely than females to change smoking behavior. Menthol status was not associated with changes in smoking among adults; however, young age, race/ethnicity, and sex were, suggesting populations to target for intervention.

## 1. Introduction

Menthol cigarettes represent a growing proportion of the cigarette market, even as overall smoking prevalence declines [1,2]. Menthol cigarette use is higher among female, non-white, and youth and young adult populations [1,3,4,5]. Evidence suggests menthol cigarettes contribute to youth cigarette smoking initiation and reduced adult cessation [6,7,8,9]. Not only may menthol contribute to smoking initiation and reduced cessation, it may also facilitate movement from lighter and infrequent smoking to heavier and more frequent smoking. Given the known health effects of continued smoking, [10] understanding what contributes to transitions in heaviness and frequency of smoking are important. This has primarily been examined in youth and young adults with studies showing associations between menthol cigarette use and transitions from infrequent, non-daily use and/or initiation to more frequent use [11,12,13,14,15]. Among youth, longitudinal studies have shown initiating menthol cigarettes (vs. non-menthol) was associated with transitions to established cigarette use and greater nicotine dependence [12,13]. Among young adults, studies demonstrated initiating with menthol cigarettes in 18–24 year olds [15] and preference for menthol cigarettes in 18–34 year olds [11] increased likelihood of subsequent cigarette use. First use of a menthol cigarette was associated with subsequent daily cigarette use in youth, young adults, and adults [14]. Among adults, research has been restricted to examining associations of initiating smoking and has not examined transitions in smoking behavior [14,15].

Given federal interest in a menthol cigarette ban [10] and calls for menthol restrictions at federal and local levels [16], it is important to understand how using menthol cigarettes is associated with patterns of cigarette use over time and if use of menthol cigarette contributes to heavier and/or more frequent smoking behavior. The existing literature is limited to smoking patterns in younger populations and there has yet to be a full study of the adult smoking population. It is unclear whether a similar relationship between menthol cigarette use and smoking progression exists in older, more established populations. The aim of this exploratory investigation was to expand upon earlier studies [11,12,13,14] by using a longitudinal adult sample to examine changes in smoking behaviors across a one-year time period.

## 2. Materials and Methods

This study used the most recent National Cancer Institute (NCI)-sponsored longitudinal Tobacco Use Supplement to the Current Population Survey (TUS-CPS) cohort data, a nationally representative survey of the United States. Wave 1 was administered in May 2010 and followed up one year later in May 2011 (Wave 2). Briefly, TUS-CPS interviews all household members 18 and older. Most were interviewed via telephone (~64%) [17] and self-response (~80%) [17]. A total of 28,153 respondents completed the May 2010–2011 longitudinal supplement, representing 67.9% of the May 2010 sample. 

Respondents were included in analyses if they were current smokers at either Wave 1 or 2, defined as established cigarette smokers (≥100 lifetime cigarettes) who reported past-30-day smoking. Of 4060 current smokers at either timepoint, 3668 had complete data on both menthol status and cigarette smoking behavior across both waves. 

Cigarette smoking behavior was estimated at each wave via self-reported: (1) average cigarettes smoked per day (CPD, range: 1–40), and (2) days smoked in the past 30-days (range: 1–30). At both waves, variables were combined to create an overall smoking value. Change in cigarette smoking behavior between waves was calculated by taking the difference in smoking behavior between waves to determine whether there was (1) maintained (i.e., no change), (2) increased, or (3) decreased current smoking behavior. For example, if someone reported 10 CPD and 10 smoking days, their overall smoking value would be ‘100′. Individuals (*n* = 251) who did not report current smoking at Wave 1, but did at Wave 2 were characterized as increasing smoking behavior; those who were current smokers at Wave 1, but not 2 (*n* = 408) were categorized as decreasing smoking behavior.

Respondents were asked about menthol status at both waves. For analyses, menthol status from Wave 1 was used ((“Do you usually smoke menthol or non-menthol cigarettes?”) and respondents denoted their menthol preference: Menthol, Non-menthol, Blank/No Preference [No Usual Type, Do not Know, Blank, No Response, Refused]). 

Given menthol preference stability over time (i.e., only 2.4% and 2.8% of Wave 1 switched to menthol or non-menthol, respectively), Wave 1 menthol status was imputed using Wave 2 data when needed using next observation carried backward. When Wave 1 menthol status was not available (*n* = 850), including those who initiated smoking at Wave 2, left menthol status blank, or had no preference at Wave 1, Wave 2 menthol status imputed to Wave 1 using baseline observation carried forward (*n* = 487). Remaining participants with “Blank/No preference” menthol status were treated as missing.

Demographics and smoking characteristics were examined by menthol status including age, sex, race/ethnicity, education, CPD, smoking frequency (i.e., every-day or some-day smoking), and time to first cigarette (TTFC) [18,19]. Multinomial logistic regressions were used to calculate relative risk ratios (RRR) for increasing and decreasing smoking behavior relative to maintaining smoking behavior by menthol status. Unadjusted models were run, followed by adjusted models, which included sex (male, female), race/ethnicity (non-Hispanic white, non-Hispanic Black, Hispanic, other race [other race combined due to sample size]), and age (18–24, 25+). Sensitivity analyses restricted the sample to 18–34 year olds to compare findings to prior young adult studies [11]. We also used to sensitivity analyses to determine if differences based on inclusion or exclusion of the “Blank/No preference” menthol status category. TUS-CPS replicate weights were applied according to recommended procedures [20]. Analyses were performed using Stata 14 software (StataCorp. College Station, TX) for complex weighted survey data.

## 3. Results

A total of 3668 participants were included in the analyses, 29.4% were menthol cigarette smokers (weighted; *n* = 977) and 70.6% (weighted; *n* = 2691) were non-menthol cigarette smokers. Menthol preference was greater in females, those reporting Black and Hispanic race/ethnicity, some-day smokers, and 18–24 year olds (Table 1). Menthol smokers had a significantly lower CPD, but no difference in TTFC (Table 1). 37.6% of the sample was categorized as maintaining smoking behavior, 36.8% as decreasing smoking behavior, and 25.6% was categorized as increasing smoking behavior.

No differences in statistical significance were observed between adjusted and unadjusted models. In the adjusted model, there was no difference by menthol preference in the likelihood of increasing (RRR = 0.91, 95% CI = 0.71, 1.17) or decreasing (RRR = 0.98, 95% CI = 0.79, 1.22) smoking behavior relative to maintaining smoking behavior. Males were less likely than females to increase (RRR = 0.79, 95% CI = 0.65, 0.96) or decrease (RRR = 0.77, 95% CI = 0.65, 0.91) smoking behavior relative to maintaining smoking behavior. Black smokers had twofold likelihood of either change in smoking behavior relative to maintaining smoking behavior compared to white smokers (RRR = 2.13, 95% CI = 1.38, 3.28 increase; RRR = 1.81, 95% CI = 1.26, 2.61 decrease). Hispanic cigarette smokers (RRR = 1.62, 95% CI 1.09, 2.40); as well as those of other ethnicities (RRR = 1.64, 95% CI = 1.11, 2.46) had greater likelihood of decreasing smoking relative to maintaining smoking compared to white smokers. Relative to older adults, 18–24 year olds were more likely to increase smoking behavior (RRR = 1.50 95% CI = 1.02, 2.19) (Table 2). 

In line with findings in the full sample, sensitivity analyses restricted to 18–34 year olds did not demonstrate differences with respect to menthol status or age and had similar findings to full analyses with regard to race and sex. Including those with menthol status as “blank/no preference” did not change our menthol status findings.

## 4. Discussion

While prior studies have addressed the influence of menthol cigarettes on smoking behavior patterns in youth/young adults, our study extended examinations into adult cigarette smokers prospectively over a one-year time period. In established smokers, we observed that males were more likely to maintain smoking behavior across time than females. We also found that, compared to white smokers, non-white smokers were more likely to change behavior over time: Black smokers were more likely to increase or decrease smoking behavior, while Hispanic and other race smokers were more likely to decrease smoking behavior. Additionally, young adults were more likely to increase smoking behavior relative to older adults.

Unexpectedly, menthol cigarette preference was not a predictor of behavior change. Our measures captured change over time among established smokers (>100 lifetime cigarettes); while this is a standard threshold for established cigarette smoking in adults, it differs from the prior work on this topic in youth and young adults, which used less restrictive measures of smoking, including past 30-day use [13], ever smoking with some past month cigarette use [12], or a 100-cigarette lifetime threshold at Wave 2 and queried Wave 1 retrospectively [11]. Taken together with prior work, our findings suggest that menthol may be a direct facilitator of change among experimental, less established populations as opposed to adult cigarette smokers with an established repertoire of smoking.

Although we did not observe differences by menthol preference, we did observe differences in rates of menthol preference by age, sex, and race/ethnicity consistent, with other national studies [1,3]. Findings regarding smoking behavior by age, race/ethnicity, and sex over time suggest opportunity within these subgroups for intervention. Young (vs. older) adults were more likely to increase smoking behavior, consistent with work demonstrating transitions in smoking occur during young adulthood [21,22,23]. Our data demonstrate that even among those considered established smokers, young adults may still be developing a pattern of smoking behavior and are more likely to have shifts in frequency and/or heaviness of smoking [24]. Focused intervention specifically among young adults may be especially important to reduce likelihood of transitioning to heavier smoking.

Consistent with prior work [11], Black smokers and females were more likely to change smoking behavior than white smokers and males. If Black smokers and females have greater likelihood of changing smoking behavior (i.e., increasing or decreasing cigarette smoking) over a relatively short time period (one year), we may be able to use this information to target smoking cessation/reduction interventions within these groups, especially given evidence that both of these subgroups have lowered success with smoking cessation [25,26,27,28,29,30,31]. Given the high prevalence of menthol use among both populations [1,3], and that they may be susceptible to changing smoking behavior, policies restricting availability of menthol cigarettes, such as the current federal proposal of a menthol ban in cigarettes [10], may harness underlying changes in smoking behavior and have greater impact on reducing smoking in these populations than previously estimated [32]. This is in line with research demonstrating restricting availability to menthol cigarettes reduced smoking rates with this effect shown to strongest among Black smokers [33]. Another consideration is to understand what contributes to behavior changes in either direction; research is needed to establish why some populations are more likely to change smoking behavior than others and how antecedents to changes in either direction may be used in interventions directed at cessation or preventing increases in smoking. Hypothetical tasks have demonstrated that availability impacts cigarette use [34,35]; these tasks may be one way to examine if the subpopulations identified by the current study have more elastic demand (i.e., use is more sensitive to change) relative to other populations. Finally, we also found that Hispanic and other race/ethnicity smokers were more likely to decrease their smoking behavior relative to white smokers, which is consistent with national cross-sectional data showing Hispanic smokers have lower smoking rates overall than white smokers [36]. These data suggest cessation efforts geared toward Hispanic smokers may be especially relevant, as this population may be susceptible to decreases in smoking.

There are some limitations to this study. The TUS-CPS captures detailed data on smoking for those with ≥100 lifetime cigarettes; it does not include inexperienced and/or experimental smokers. Second, data were collected between 2010–2011; thus, there were no questions investigating tobacco products after that time period. While these data do not include more recent products, it is important to note that the patterns observed would still be likely to occur in an environment with newer tobacco products. Future research should examine whether this pattern persists among current tobacco users and if product switching occurs in groups that demonstrate changes, specifically decreases, in smoking behavior. Additionally, respondents without reported menthol status at either timepoint were excluded from analyses. While inclusion of the “Blank/No preference” category in models did not impact findings, this may reduce model estimates’ precision.

## 5. Conclusions

In conclusion, menthol cigarette preference was not a correlate of change in smoking behavior over a one-year time period among established adult cigarette smokers. Female, young, and non-white populations were more likely to change than maintain smoking behavior over one year. These findings suggest specific interventions to aid smoking cessation may be relevant for these groups with more transient smoking behavior. Broader population-level approaches to make cigarettes less appealing, affordable, and available hold promise and may be a way to facilitate smoking reduction and reduce tobacco-related health disparities in U.S. communities [37].

## Figures and Tables

**Table 1:** Sociodemographic of smoking characteristics by menthol preference at Wave 1.

	Menthol Preference		Non-Menthol Preference		*p* Value
	(*n* = 977)		(*n* = 2691)		
	Percent	95% CI	Percent	95% CI	
Sex					<0.001
Male	23.6	(21.3, 26.0)	76.4	(74.0, 78.7)	
Female	36.0	(33.5, 38.5)	64.0	(61.5, 66.5)	
Age Group					<0.001
18–24 years	41.9	(34.6, 49.5)	58.1	(50.5, 65.4)	
25+ years	28.0	(26.2, 29.8)	72.0	(70.2, 73.8)	
Race/ethnicity					<0.001
White, NH	22.3	(20.5, 24.1)	77.7	(75.9, 79.5)	
Black, NH	74.9	(69.7, 79.3)	25.1	(20.6, 30.3)	
Hispanic/Latino	32.6	(26.9, 38.9)	67.4	(61.1, 73.1)	
Other, NH	26.8	(19.3, 36.0)	73.2	(64.0, 80.7)	
Education					0.25
Less than High School	31.0	(26.6, 35.8)	69.0	(64.2, 73.4)	
High School Degree/Equivalent	30.0	(27.4, 32.6)	70.0	(67.4, 72.6)	
Some college	29.8	(26.7, 33.1)	70.2	(66.9, 73.3)	
College Graduate	24.4	(19.5, 30.2)	75.6	(69.8, 80.5)	
Smoking Days					0.02
Every-day	27.9	(25.9, 29.9)	72.1	(70.1, 74.1)	
Some-day	33.6	(29.4, 38.2)	66.4	(61.8, 70.6)	
Time to first cigarette					0.26
Less than 30 min	27.3	(25.1, 29.8)	72.7	(70.3, 74.9)	
30 min or more	29.6	(26.3, 33.2)	70.4	(66.8, 73.7)	
	Mean	95% CI	Mean	95% CI	
Cigarettes Per Day	14.2	(13.5, 14.9)	16.5	(16.1, 16.9) <0.001	
Past 30-day Smoking Days	26.8	(26.2, 27.4)	27.3	(27.0, 27.6) 0.13	

Data are presented as row percentages. Chi-square tests were conducted for categorical variables and independent sample *t*-tests for continuous variables.

**Table 2:** Relative risk ratio for increasing or decreasing smoking behavior relative to maintaining smoking behavior over a 12-month period.

Increased Smoking Behavior from May 2010 to May 2011 ^a^			Decreased Smoking Behavior from May 2010 to May 2011 ^a^		
	RRR	95% CI		RRR	95% CI
Cigarette Preference			Cigarette Preference		
Menthol Preferring	0.91	(0.71, 1.17)	Menthol Preferring	0.98	(0.79, 1.22)
Non-Menthol Preferring	1.00	ref	Non-Menthol Preferring	1.00	ref
Sex			Sex		
Male	**0.79**	**(0.65, 0.96)**	Male	**0.77**	**(0.65, 0.91)**
Female	1.00	ref	Female	1.00	ref
Age group, years			Age group, years		
18–24	**1.50**	**(1.02, 2.19)**	18–24	1.13	(0.77, 1.67)
25+	1.00	ref	25+	1.00	ref
Race/ethnicity			Race/ethnicity		
Black, NH	**2.13**	**(1.38, 3.28)**	Black, NH	**1.81**	**(1.26, 2.61)**
Hispanic/Latino	**1.62**	**(1.09, 2.40)**	Hispanic/Latino	**1.53**	**(1.05, 2.23)**
Other, NH	1.08	(0.64, 1.84)	Other, NH	**1.65**	**(1.11, 2.46)**
White, NH	1.00	ref	White, NH	1.00	ref

**Bolded** values represent a statistically significant finding (*p* < 0.05). ^a^ Relative to maintaining smoking. NH = Non-Hispanic. 25+ = In the dataset, age is entered as a continuous variable until age 79 and a categorical variable from those 80–84 and 85+.

## Data Availability

The data presented in this study are openly available in https://cancercontrol.cancer.gov/brp/tcrb/tus-cps/questionnaires-data (last accessed on 15 July 2021).
